# Reception of patients admitted to a psychiatric unit

**DOI:** 10.1192/j.eurpsy.2021.1337

**Published:** 2021-08-13

**Authors:** H. El Kefi, I. Gafsi, M.L. Biloa Molo, W. Krir, C. Bencheikh Brahim, A. Oumaya

**Affiliations:** 1 Psychiatric Unit, Military Hospital of Tunis, tunis, Tunisia; 2 Psychiatry, military medical school, tunis, Tunisia

**Keywords:** patient admission, quality of health care

## Abstract

**Introduction:**

The reception of a patient in the psychiatric ward is an important step that determines the proper course of care. The welcome is the first stage of the relationship, it is essential to take the measure of the importance of this moment.

**Objectives:**

Assess psychiatric inpatients’ knowledge of their rights and obligations.

**Methods:**

This was a descriptive and cross-sectional study based on a self-administered questionnaire containing about twenty questions (20), which assessed the knowledge of patients hospitalized in the HMPIT psychiatry department about their rights and obligations.

**Results:**

Twenty-five (25) patients agreed to answer the questionnaire and two (2) patients refused. Sixty percent (60%) of the patients were unaware of their rights. Sixty-eight percent (68%) of the patients did not know their duties. Forty-eight percent (48%) of the patients did not know the rules and conditions of hospitalization in psychiatry.

**Conclusions:**

Patients hospitalized in the psychiatric ward have limited knowledge about their rights and duties and about the conditions and rules governing hospitalization. In addition to the poster on patients’ rights and duties, a welcome leaflet will provide all the information on the rules of hospitalization.

